# The Beauty Sleep to Keep a Healthy Liver

**DOI:** 10.3390/ijms262311322

**Published:** 2025-11-23

**Authors:** Mariana Verdelho Machado

**Affiliations:** 1 Clínica Universitária de Gastrenterologia, Faculdade de Medicina, Universidade de Lisboa, 1649-028 Lisboa, Portugal; mverdelhomachado@gmail.com; 2 Unidade de Oncologia Digestiva, Fundação Champalimaud, 1400-038 Lisboa, Portugal; 3 Serviço de Gastrenterologia, Hospital de Vila Franca de Xira, 2600-009 Vila Franca de Xira, Portugal

**Keywords:** liver disease, cirrhosis, MASLD, sleep disorders

## Abstract

Sleep disturbances and liver diseases have a bidirectional relationship. Unhealthy sleep habits promote liver diseases, such as steatotic liver disease, and impact the prognosis, promoting progression to liver cirrhosis and liver-related mortality. Sleep accounts for 20% of the association between lifestyle and steatotic liver disease, indirectly by promoting obesity and metabolic syndrome and through direct effects in the liver. Conversely, liver diseases can affect sleep. Patients with liver cirrhosis complain of sleep disturbances five times more than the general population, with a profound impact on their quality of life. Common drugs used to treat sleep disorders, such as hypnotics and benzodiazepines, must be used very carefully in patients with cirrhosis due to altered hepatic metabolism and the potential to induce hepatic encephalopathy, making sleep disorders particularly challenging to manage in these patients. This review summarizes the available knowledge on the interplay between sleep and liver diseases.

## 1. Introduction

Sleep is a biological requirement, as is the air we breathe, the water we drink, and the food we eat [1]. Humans spend between 20 and 40% of their day sleeping [1]. If sleep did not serve a vital physiological function, it would be a waste of time. Indeed, studies in rodents (rats) have shown us, more than 50 years ago, that extreme sleep deprivation can be lethal [2]. We know today that insufficient sleep not only impacts cognitive function and psychiatric health but also affects the development of weight gain and obesity, metabolic syndrome (MS), type 2 diabetes mellitus (T2DM), accidents, immune dysfunction, cardiovascular diseases, cancer, and even mortality [3].

Sleep disorders can be divided into seven major categories: (1) insomnia, (2) sleep-related breathing disorders, (3) central disorders of hypersomnolence, (4) circadian rhythm sleep–wake disorders, (5) sleep-related movement disorders, (6) parasomnias, and (7) other [4].

Sleep hygiene is an important health lifestyle behavior that can prevent morbidity and mortality. There is popular knowledge regarding the importance of quality sleep, which is demonstrated in common expressions such as “beauty sleep”. However, public policies, medical practice, and even medical education do not pay proper attention to it. Illustrating the underrating of sleep health, a worldwide survey of 400 medical schools showed that the average time spent on education on sleep was just 2.5 h, and one-fourth of medical schools did not even approach the topic [5].

US health surveys have shown that one-fourth of the population sleeps less than the desired 7 h per night [6], and that 10% report sleep disturbances [7]. However, studies show a 0.1% prevalence rate of sleep disorders in community-based outpatient health settings and 3% in university-based clinics, which highlights the impressively low rate of recognition and diagnosis of sleep disorders [8].

Liver diseases are associated with a particularly high prevalence of sleep disorders. For example, they have been described in one-third of patients with metabolic dysfunction-associated steatotic liver disease (MASLD) [9], half of the patients with primary biliary cholangitis [10], two-thirds of patients with chronic hepatitis C [11], and up to 80% of patients with liver cirrhosis [12]. The relationship between sleep and liver disease is complex and bidirectional. Sleep behavior can promote liver disease, particularly MASLD, and increase the risk of progression to liver cirrhosis and liver-related mortality. Other liver diseases present specific sleep disorders that can be seen as extrahepatic manifestations, such as Wilson’s disease. Additionally, the effect of liver cirrhosis on sleep can have an important impact on the quality of life of these patients.

This review critically summarizes the interplay between sleep behavior, sleep disorders, and liver diseases that all hepatologists should be aware of (Table 1).

## 2. Sleep Physiology Basics

The rotativity between asleep and awake states depends on the interplay between arousal-stimulating noradrenergic nuclei of the brainstem, hypocretinergic neurons of the lateral hypothalamus that stabilize the wake state, and sleep-promoting galaninergic or GABA nuclei of the basal forebrain (BF), such as the hypothalamic ventrolateral preoptic area (VLPO) and median preoptic nucleus (MnPN) [13].

Sleep is highly regulated by two main processes: homeostatic (process S) and circadian pacemakers (process C) [14,15,16]. The homeostatic process reflects the increasing need to sleep with an increasing duration of the waking period or cumulative sleep debt. In contrast, as we sleep, the need to sleep progressively decreases [15,17]. As such, process S acts as a “somonstat” ranging between two thresholds of sleep debt and sleep needs being fulfilled [18]. Circadian clocks synchronize the sleep–wake cycle to the 24 h day–night cycle [19], prevailing over the homeostatic process that monitors internal demands. However, when sleep pressure is very high, it abrogates circadian rhythm control [18]. Misaligned homeostatic and circadian control, as a result of shifted schedules, jet-leg, or sleep disorders, are detrimental to health [20], including liver health.

The homeostatic process translates into an exponential decline in slow-wave activity during sleep [18]. The level of slow-wave activity at the beginning of the sleep episode is determined by the sleep history. For example, sleep deprivation intensifies slow-wave activity in the “recovering night”, whereas a daytime nap attenuates it in the subsequent night sleep episode [21]. A biochemical marker of the homeostatic process is adenosine [20]. As the waking period progresses, brain adenosine levels increase, whereas during sleep, adenosine levels decrease [22]. Caffeine, an adenosine receptor antagonist, is a well-known inhibitor of sleepiness [23].

Adenosine acts by binding to G-protein-coupled receptors, two of which are implicated in sleep regulation: A1R and A2AR [24]. Both adenosine receptors play different roles in sleep regulation. A1R is widely expressed in the brain and is the receptor with the highest affinity. Presynaptic A1R binding inhibits the adenylyl cyclase (AC)-cAMP-dependent protein kinase pathway, whereas postsynaptic binding opens potassium channels, resulting in neuronal membrane hyperpolarization, reducing excitability, and promoting neuroprotection. A1R effects are dependent on the brain region. A1R can induce sleep by inhibiting cholinergic and non-cholinergic wakefulness-promoting basal forebrain (BF) neurons [25]. A1R partially inhibits hypocretin neurons in lateral hypothalamic peptidergic neurons, promoting sleep [26]. Furthermore, adenosine binding to A1R inhibits histamine release from the tuberomammillary nucleus, preventing histamine-mediated NREM sleep termination and hence prolonging NREM sleep [27]. A2AR expression in the brain is restricted to specific regions. A2AR activates type 2 GABAergic neurons in two hypothalamic regions, the VLPO and MnPN, promoting the initiation of sleep by inhibiting arousal systems [28], and neurons in the nucleus accumbens (NAc) triggering slow-wave sleep [29]. A2AR signals through the activation of adenylate cyclase, among other mechanisms.

Adenosine levels depend on its production and the removal of extracellular adenosine into cells through nucleoside transporters [30]. Adenosine is produced through the dephosphorylation of neurons- and astrocytes-derived ATP to AMP, and from AMP to adenosine by 5′-nucleotidases. Conversely, high adenosine levels can be phosphorylated to AMP through the action of adenosine kinase, whose expression in glial cells is controlled by the metabolic state [24,30]. Indeed, adenosine links sleep to energy metabolism and neuronal activity. During wakefulness, the high demand for ATP increases adenosine production and sleep propensity [31]. In a way, adenosine levels represent a state of relative energy deficiency [30].

Sleep is under the regulation of circadian clocks in virtually all animals, although the underlying mechanisms may be species-specific [32]. The circadian rhythm is controlled by clocks organized in a hierarchical system, with the suprachiasmatic nuclei (SCN) of the anterior hypothalamus acting as the dominant central clock in mammals, while virtually every tissue contains subsidiary peripheral clocks. These clocks respond to several external and internal cues, the *zeitgebers* [19]. The most important cue is light [20], which is perceived by the intrinsically photoreceptive retinal ganglion cell (ipRGC) expressing the photopigment melanopsin that delivers information to the SCN through the retinohypothalamic tract [33]. Other stimuli can modulate the rhythm even without direct action in the clocks, such as light at night, physical activity, and the timing of meals [34].

Circadian rhythms are generated, at the molecular level, through complex feedback loops of transcription of clock genes, which are highly conserved across eukaryotes, including *Drosophila*, zebrafish, and mammals [35]. The maestros are circadian locomotor output cycle kaput (CLOCK) and brain and muscle ARNT-like 1 (BMAL-1) [36], which form heterodimers that bind to the DNA cis-element E-box and act as transcription factors for 1500–3000 target genes, as shown in a genome-wide analysis in mouse liver [37,38,39]. Examples of target genes are the gene families period (PER-1, -2, and -3) [40] and cryptochrome (CRY-1 and -2), transcription promoters retinoic acid-related orphan nuclear receptors (ROR-α, -β, -γ) and albumin D-element binding protein (DBP)/hepatic leukemia factor (HLF), transcription repressors REV-ERB-α, -β, and the basic helix–loop–helix proteins differentiated embryo chondrocytes (DEC-1, -2) [41]. In turn, PER and CRY heterodimers are transcription repressors that can inhibit CLOCK-BMAL-1 expression, thereby regulating their own expression [35]. RORs and REV-ERBs also modulate BMAL-1 expression [42]. DBP/HLF promote the transcription of several genes by binding to the DNA-cis-element (D-box) during the rest phase. In the active phase, DBP/HLD transcription activity is repressed through competitive D-box binding by the E-4 promoter binding protein 4 (E4BP4) repressor, the expression of which is regulated by REV-ERBs. The DBP/HLF and E4BP4 interplay helps maintain the circadian rhythm; however, E4BP4 responds to other stimuli, such as insulin, glutamate, and reactive oxygen species, and can induce circadian phase shifts and clock resetting [43]. Finally, DEC proteins compete with CLOCK-BMAL-1 for the promoter of its target genes, hence suppressing the expression of all their target genes [44].

Regarding circadian sleep regulation, in mammals, most of the SCN output goes to the subparaventricular zone (SPZ) to be integrated in the dorsomedial nucleus of the hypothalamus (DMH), with information regarding food availability, visceral sensory inputs, cognitive influences from the prefrontal cortex, and emotional inputs from the limbic system [13]. DMH seems to promote wakefulness through projections into the VLPO from GABA-containing neurons (that inhibit sleep) and into orexin neurons in the lateral hypothalamus (LHA) from excitatory glutamate-containing neurons (stabilizing wakefulness) [45].

Peripheral clocks also seem to actively participate in the regulation of sleep, in a more species-specific fashion. For example, muscle clocks seem to regulate the amount of sleep and promote restorative sleep [46]. Indeed, experiments in mice showed that muscle BMAL-1 regulates peroxisome proliferator-activated receptor gamma coactivator 1-alpha (PGC1α), which upregulates irisin expression, an inducer of brain-derived neurotrophic factor (BDNF) [46]; and that BDNF promotes restorative slow-wave activity during sleep [47]. In humans, exercise increases BDNF production (through an increase in the ketone body D-β-hydroxybutyrate that increases BDNF [48] expression through histone deacetylation [49]) and improves sleep quality [50]. Food intake and energy metabolism also have a strong effect on sleep regulation [51], as increased feeding and high-calorie meals lead to excessive sleep [52], whereas starvation suppresses sleep [53]. The influence of feeding on sleep is, at least in part, mediated by the vagus nerve, which contains both afferent and efferent fibers that allow for bidirectional communication between the brain, the gastrointestinal tract, and the liver. Recent studies in mice showed that the vagus nerve mediates a brain–liver axis that regulates metabolism, such that disruption of vagal innervation of the liver promotes glycolysis and lipogenesis while decreasing fatty acids oxidation, resulting in increased susceptibility for liver steatosis [54]. In turn, the disruption of liver circadian regulation (either genetically or through a high-fat diet), alters feeding behavior, leading to increased intake during the resting phase, a process also mediated by the vagus nerve [55]. In rodents, a high-fat diet is also associated with reduced wakefulness and altered sleep architecture [51,56,57,58]. In humans, a high-fat diet slows the adaptation of sleep–wake timing to changes in the time schedule with an hour’s anticipation for daylight-saving time [59]. How the liver acts as a “metabolismostat” and modulates sleep physiology remains an area that warrants further investigation.

Biochemical markers of the circadian rhythm include melatonin, cortisol (which rises in the morning in response to light), and growth hormone (which is secreted during deep sleep) [16]. In humans, in response to light and darkness, the SCN acts on the pineal gland through the sympathetic system, resulting in an increase in melatonin secretion soon after the onset of darkness, peaking in the middle of the night, and progressively declining thereafter [16,20]. The nocturnal rise in melatonin promotes the propensity to sleep, but is suppressed by exposure to light [17]. Notably, melatonin is not essential for circadian regulation, since some mammals do not produce melatonin and still have a circadian regulation of sleep, such as many mouse strains, for example the C57Bl/6J strain [60].

Sleep is a cyclic process that can be divided into rapid eye movement (REM) and non-REM (NREM) sleep, which can be subdivided into three stages N1–N3 [16,61]. Each stage can be differentiated using an electroencephalogram (EEG). During active wakefulness, the EEG is characterized by very-high-frequency γ oscillations (at least 40 Hz) and desynchronized β activity (approximately 20–30 Hz), whereas relaxed wakefulness presents synchronized α activity (approximately 10 Hz). Stage N1, a transition between wakefulness and sleep, is characterized by θ activity (4–7 Hz) and corresponds to approximately 5% of a night’s sleep. Stage N2 is a light sleep when the eyes become still, the heart rate slows, the body temperature decreases, and memory consolidation occurs. The EEG is characterized by sleep spindles (of 12–14 Hz lasting up to 1 s) and K complexes (high-amplitude biphasic waves). N2 corresponds to approximately 50% of the night’s sleep. Stage N3, deep sleep or slow-wave sleep (SWS), is characterized by high-amplitude slow δ waves (0.3–2 Hz). At this stage the muscles are totally relaxed, and this corresponds to 20–50% of the night’s sleep. Finally, REM sleep is when most dreaming occurs and memory consolidation occurs. Rapid eye movement contrasts with arm and leg skeletal muscle atony, preventing dreams from translating into actions. Breathing and heart rate also increase. EEG is characterized by desynchronized neural activity, with slow α (1–2 Hz) and θ waves. REM sleep corresponds to approximately 20% of a night’s sleep [16,62].

Sleep architecture refers to the proportion of each stage of sleep [63]. An 8 h night sleep is structured in 4–5, 90 min, cycles of N1 to N3-REM sleep. As the evening progresses, stage 2 increases its predominance in NREM, and REM becomes longer [16,62]. N3 δ activity is an indicator of sleep homeostasis, with this activity increasing in proportion to sleep needs [64], whereas REM sleep is under circadian control and homeostatic process inhibition [18].

Although the function of sleep remains unknown [20], it plays an important role in brain function, such as cognitive performance and memory consolidation, as well as in maintaining cardiovascular health, immune system function, and hormonal and metabolic regulation [16,65].

## 3. How Can We Study Sleep Disturbances?

Sleep can be evaluated subjectively using standardized sleep diaries [66] or questionnaires. The most validated questionnaire is the Pittsburgh Sleep Quality Index (PSQI), which assesses 19 questions across seven components referring to the previous month: (1) sleep quality; (2) sleep onset latency (SOL); (3) total sleep time (TST); (4) sleep efficiency; (5) sleep disturbances; (6) sleep medication; and (7) daytime dysfunction. Each component is scored 0 to 3, with the higher scores indicating poorer sleep quality. A PSQI of 5 or higher is considered indicative of poor sleep [67]. The PSQI is the gold-standard self-administered tool, but it requires approximately 10 min to complete and an additional 5 min to score [68]. Simpler and less time-consuming tools such as the Sleep Timing and Sleep Screening Questionnaires (STSQS) have been validated in patients with cirrhosis, showing a good correlation with the PSQI [69].

There are also subjective tools to evaluate the repercussions of poor sleep quality, namely excessive daytime somnolence (EDS), with the most validated being the Epworth Sleepiness Scale (ESS) [70]. The ESS assesses the likelihood of falling asleep in daytime situations and yields a total score ranging from 0 to 24. Higher scores indicate greater sleepiness, and a score of 11 or more is considered abnormal [68].

Sleep can also be evaluated objectively, with polysomnography (PSG) being the gold-standard tool. PSG requires a laboratory setting, specialized equipment, and expertise, and it monitors, during sleep, brain EEG, eye movements, skeletal muscle activity, blood oximetry, and heart and breathing rhythms. It allows for an objective evaluation of SOL, TST, wake time after sleep onset (WASO), number of awakenings, and sleep efficiency. It also provides detailed EEG monitoring, in which altered sleep architecture and a high-to-low frequency ratio are indicators of sleep quality, with greater ratios indicating poorer sleep [63].

Actigraphy is a semi-quantitative technique, less expensive than PSG and capable of being performed at the patient’s home. It requires an actigraph, a device resembling a wristwatch that monitors movement. It relies on the assumption that movement corresponds to wakefulness and lack of movement to sleep [68]. More recently, researchers have been using commercial consumer wearable sleep-tracking devices, which offer cheaper larger-scale availability but must be interpreted with caution, as they were not developed for clinical or research purposes and are not properly validated [71].

## 4. How Can Sleep Disturbances Promote MASLD?

MASLD affects one in three people worldwide [72], being the leading cause of chronic liver disease in the Western countries [73]. It is also a growing cause of end-stage liver disease, already ranking as the leading indication for liver transplantation in women and the second one in men in the US [74]. MASLD is associated not only with liver-related mortality but also with increased overall mortality compared to the general population, with cardiovascular diseases and cancer being the leading causes of death [75].

Although there seems to be a genetic susceptibility to the development and progression of MASLD [76], it is mostly associated with obesity and adiposopathy, T2DM, and MS [77]. Behavioral factors have a huge impact on the development of metabolic dysfunction, obesity, and MASLD, and sleep quality seems to account for up to 20% of the association between lifestyle and MASLD [78].

MASLD is consistently associated with sleep disturbances in human studies. Small case–control studies with well-characterized MASLD patients [9,79,80,81], have shown that MASLD is associated with subjectively assessed shorter sleep duration, increased sleep latency, poor sleep quality (by PSQI), frequent arousals, and a shift in food intake toward the night. MASLD has also been linked to more frequent awakenings and wakefulness after sleep, fragmented sleep—particularly fragmented REM sleep (resulting in less restorative sleep)—and lower sleep efficiency, as assessed by actigraphy and PSG. Steatohepatitis/hepatic inflammation seems to be associated with more severe sleep disorders than isolated steatosis [79,82,83], although data remain limited. Advanced fibrosis and liver cirrhosis are also associated with poorer sleep quality, lower sleep efficiency, and an increasing number of secondary sleep episodes [9,84]. Notably, sleep disturbances and sleep quality predicted up to 20% of the variability in liver stiffness [80]. Daytime sleepiness (by ESS) was associated with insulin resistance (IR), liver enzyme elevation, and most importantly, advanced liver fibrosis [79].

Large cross-sectional epidemiological studies have also identified positive associations between MASLD prevalence and extreme sleep durations, sleep duration intraindividual day-to-day variability, irregular sleep onset times, poor sleep quality, increased sleep latency, evening chronotype, difficulty in waking up in the morning, snoring, insomnia, narcolepsy, and excessive daytime sleepiness [85,86,87,88,89,90,91,92,93,94,95]. Studies using Mendelian randomization have identified overlapping genetic variants associated with poor sleep quality, insomnia, snoring, and daytime sleepiness with variants associated with MASLD, providing genetic evidence supporting causality [96]. Long-term longitudinal epidemiological studies further support a potential causal role of sleep disturbances in incident MASLD [97]. Liver fibrosis and cirrhosis have also been positively associated with poor sleep quality, snoring, sleep apnea, and excessive daytime sleeping [95,98].

An association between sleep duration and MASLD prevalence or incidence has been extensively investigated in epidemiological studies [86,88,93,99,100,101,102,103,104,105]. Aggregate data suggest a U-shape curve for sleep duration, with a nadir between 7 and 8 h sleep, and risk of MASLD, liver fibrosis and cirrhosis, hepatocellular carcinoma, and liver-related mortality [88,93,105]. The impact of sleep duration on MASLD risk presents gender differences, being more pronounced in men, and seems to be modulated by body mass index (BMI) [91,100,101,106]. This U-shape curve resembles the known U-shape association between sleep duration and MS, IR/T2DM [107,108,109], and all-cause and cardiovascular mortality [110]. Short sleep can promote obesity and MS through circadian misalignment (see other reviews [19]) and direct effects of sleep deprivation [111]. Sleep deprivation induces a mild increase (5–7%) in energy expenditure, reflecting the energy cost of prolonged wakefulness [112]. However, sleep deprivation leads to a disproportionately higher increase in energy intake (estimated at around 250 kcal per day) [113] during the evening and the day after, mainly from high-calorie, high-fat, and high-carbohydrate foods [112], and from snacks rather than meals [114]. This increased energy intake may result from elevated subjective hunger driven by a higher hunger hormone ghrelin to satiety hormone leptin ratio [115,116]. Indeed, sleep deprivation blunts the inhibitory effect of sleep on ghrelin levels in the second half of the night (which helps to suppress overnight hunger in normal sleep) and is associated with lower leptin levels [111,117]. Furthermore, sleep deprivation enhances hedonic food drive through altered brain activity in reward-related regions, such as the amygdala and hypothalamus [118,119,120]. The phases of sleep seem to affect food intake differently, since REM-deprived rats present hyperphagia and hypoleptinemia [121]. Sleep deprivation may also promote weight gain through altered DNA methylation in adipose tissue and skeletal muscle, promoting an anabolic signature in the former and a catabolic signature in the latter [122]. Finally, sleep deprivation may promote insulin resistance via hypercortisolism (which is dependent on circadian regulation), increased growth hormone secretion (which is sleep-dependent), and systemic inflammation [103,123,124,125].

Weekend catch-up sleep (WCUS) to compensate for short sleep duration during the week seems beneficial, as large epidemiological studies have shown it to neutralize the increased risk for MASLD associated with short weekday sleep [126]. WCUS has similar protective effects on BMI (with every additional hour of WCUS resulting in a 0.12 kg/m^2^ decrease in BMI) [127], hypertension [128], and MS [129].

In contrast, napping does not seem beneficial. Indeed, meta-analyses showed that napping, especially for longer than 30 min, was associated with a dose-dependent increased risk for MASLD (about 40% for 30 min and 80% for 60 min naps) [130]. The impact of napping synergizes with poor sleep quality [131]. Napping has also been linked to a linearly increased risk for all-cause mortality and a J-shaped association with cardiovascular disease risk, with mild benefits only for naps shorter than 30 min and sharp increases beyond 45 min [132,133,134]. Longer naps (longer than 1 h) were associated with obesity [135] and T2DM [136]. Mechanistically, short naps end before slow-wave sleep begins, whereas longer naps enter but do not complete it. Naps are dominated by NREM sleep, while WCUS restores REM sleep. Napping may thus result in circadian misalignment, while WCUS represents a circadian-aligned extension of natural sleep. Moreover, napping is often associated with pathological sleep (for example, obstructive sleep apnea, [OSA]), whereas WCUS reflects recovery from sleep debt [126].

Regarding late-chronotype, a delayed bedtime after 10 pm was associated with a 20% increased MASLD risk, and a bedtime after midnight with up to 40% higher risk [89,94,137]. This risk persists even with normal sleep duration but is exacerbated by short sleep and decreases by 8% per additional hour of sleep [138]. Bedtime sleep after midnight was also associated with a 2.5-fold higher risk of advanced liver fibrosis [139,140], as well as with an up to 30% increased risk of hypertension, 70% risk of MS, and 2.5-fold higher risk for T2DM, independent of sleep duration or efficiency [141,142,143]. These associations are partly explained by the unhealthier lifestyle among late-chronotypes: later meal times, higher caloric intake at dinner, greater consumption of fast food and soft drinks with lower fruit and vegetables intake [144], smoking, alcohol use, higher exposure to artificial light at night, and misalignment between social and circadian rhythms [143,145].

Preclinical studies in animal models suggest a causal link between sleep disturbances and MASLD development. In mice, sleep deprivation is both diabetogenic and steatogenic, promoting hepatic lipogenesis and reducing lipolysis [146]. REM sleep deprivation in mice increases susceptibility to steatohepatitis, resulting in higher liver enzymes and proinflammatory cytokines levels [147]. Lastly, mice that were clock-deficient or submitted to constant darkness, with impaired circadian regulation, exhibited a blunted circadian activity and feeding rhythm, with no differences in food intake between light and darkness periods. Interestingly, those mice were hyperphagic, obese, and developed IR, MS, and MASLD [148,149].

Attempts to mitigate the effect of poor sleep quality on MASLD risk through other healthy lifestyle interventions have shown mixed results. Physical activity does not appear to abrogate the risk [150], but a fiber-rich diet may attenuate it [151].

An association between obesity-related MASLD and obesity-related OSA is intuitive. Indeed, extensive research, confirmed by multiple meta-analyses [82,152,153,154,155,156], found independent positive associations between OSA and higher aminotransferases levels, up to 3-fold increased risk of MASLD (by ultrasound and histology), steatohepatitis, and a 2.5-fold higher risk of liver fibrosis. Strong dose-dependent associations with MASLD have also been described for snoring [131,157].

OSA is a breathing sleep disorder characterized by episodic upper airway collapse during sleep, leading to intermittent hypoxia, hypercapnia, and sleep fragmentation, with systemic dismal consequences [158]. It affects about one-third of middle-aged men and up to one-fifth of women [159].

Mendelian randomization studies suggest a potential causal effect of genetically predicted OSA on MASLD risk [160]. OSA and intermittent hypoxia induce oxidative stress, mitochondrial injury [161], and chronic systemic inflammation [162,163], contributing to widespread tissue injury, including liver injury [164,165]. Hypoxia-inducible factors (HIF) are directly steatogenic in hepatocytes (promoting lipogenesis, increasing fatty acids uptake, and reducing lipolysis) and are both proinflammatory and profibrogenic [166,167,168,169]. Intermittent hypoxia also favors IR and T2DM [170,171] through sympathetic activation [172], hypothalamic–pituitary–adrenal axis stimulation [173], adiposopathy [174,175,176], and pancreatic injury [177], as well as increased circulating free fatty acids (particularly saturated fatty acids), which will reach the liver, leading to the build-up of hepatic steatosis. Chronic intermittent hypoxia also induces gut dysbiosis [178,179,180,181] and gut barrier dysfunction [182,183], which further exacerbates IR, MS, and MASLD [184].

Despite the strong association between OSA and MASLD, the impact of OSA treatment with continuous positive airways pressure (CPAP) remains uncertain. A meta-analysis of five small randomized controlled trials with 251 participants submitted to short-term CPAP therapy (4 to 12 weeks) failed to show improvements in aminotransferases levels [185]. However, a small long-term trial with CPAP treatment for 2 to 3 years did achieve an improvement in liver enzymes and hepatic steatosis in compliant patients [186], suggesting that prolonged treatment may be required to reverse OSA-induced liver injury.

## 5. Interplay Between Sleep Disorders and Other Liver Diseases

### 5.1. Sleep Disorders as an Injury Mechanism Induced by Alcohol

Alcohol intake and sleep/circadian regulation exert bidirectional effects on each other [187].

Both acute and chronic alcohol consumption may promote circadian misalignment. Rodents given access to alcohol exhibit altered circadian activity rhythms, particularly when alcohol is administered compulsorily rather than voluntarily [188,189,190,191]. In rodents, both acute and chronic alcohol exposure also impair the circadian pacemaker’s response to light stimulus [190,191,192].

Acute alcohol intake initially acts as a sedative, as it functions as a GABA agonist [193], reducing sleep-onset latency, increasing slow-wave sleep, and decreasing REM sleep during the first half of the night. However, as the night progresses and blood alcohol concentration declines, sleep is disrupted, with increased wakefulness and stage N1 NREM, as well as delayed REM onset and reduced REM duration [194]. Patients with alcohol use disorder (AUD) typically present with insomnia, excessive daytime sleepiness, and disrupted sleep architecture, characterized by reduced slow-wave activity and increased proportions of N1 NREM and REM sleep [195]. Moreover, these patients display blunted homeostatic recovery following sleep deprivation, evidenced by the absence of the normal rebound increase in slow-wave sleep during the recovery night [196,197]. Acute alcohol intake inhibits adenosine reuptake via the equilibrative nucleotide transporter-1 (ENT-1), whereas chronic intake downregulates ENT-1 expression, thereby enhancing adenosine’s effects. Indeed, adenosine actions are similar to the effect of alcohol, facilitating NREM sleep, increasing delta frequency power, and suppressing REM sleep [31].

Even after alcohol cessation, sleep disturbances persist. While a partial improvement is observed after 1 year of abstinence, sleep does not fully normalize even after 2 years [198]. In the abstinent, sleep tends to remain shorter, fragmented, and superficial, with prolonged sleep latency and a lower proportion of N3 NREM sleep, indicating reduced sleep efficiency. Alcohol withdrawal increases hepatic tryptophan metabolism, with subsequent low cerebral serotonin levels, which may contribute to persistent sleep disturbances in abstinent patients [199]. The severity of post-abstinence sleep disturbances predicts future alcohol relapse. For example, insomnia and sleep fragmentation persisting at 5 months of abstinence predict relapse within one year [198]. Likewise, a high proportion of REM and low N3 sleep after one month (reflecting elevated central nervous system arousal), predicts relapse within 6 months [199].

Circadian misalignment may also predispose to alcohol use. For example, drinkers more frequently tend to be late chronotypes [200], and late chronotypes drink roughly twice as much alcohol as early chronotypes [201]. Childhood overtiredness has also been associated with later alcohol use and alcohol-related problems in young adulthood [202]. Humans carrying genetic variants that regulate the circadian rhythm show greater vulnerability to heavy drinking and alcohol use disorder [203,204,205,206]. Preclinical models support this association. In *Drosophila melanogaster*, sleep deprivation induces binge-like alcohol responses and increases alcohol-induced mortality [207]. In mice, both alcohol preference and intoxicating effects follow a circadian rhythm, which is lost in *Per2*-deficient mice with circadian deregulation [208,209]. Similarly, selectively bred mice with high alcohol preference display altered circadian activity rhythms [190,210]. Mice with disrupted circadian systems, either genetically or environmental (via manipulation of light/darkness rotativity), change their alcohol preference and total alcohol intake [211,212,213,214,215].

### 5.2. Sleep Disturbances as an Extrahepatic Manifestation of Primary Biliary Cholangitis (PBC)

A cardinal symptom of PBC is fatigue, which is independent of the severity of liver disease but correlates with sleep quality [216], suggesting a shared central pathogenic mechanism. One possible explanation involves changes in cerebral proinflammatory cytokines, such as IL-6, which not only contributes to fatigue but also plays a role in the regulation of circadian sleep patterns [217].

Approximately half of PBC patients experience sleep disturbances, a prevalence more than 2.5-fold higher than that of the general population. Sleep disturbances are associated with worse liver biochemistry and poorer response to treatment [10]. Compared with healthy controls, patients with PBC typically show poorer sleep quality, longer sleep-onset latency, and shorter sleep duration, as well as a fivefold increase in excessive daytime sleepiness and twice as much daytime sleep time. Sleepiness tends to be most pronounced in the morning and early afternoon [218,219]. Up to 30% of PBC patients also present with restless leg syndrome [217].

Pruritus has a major impact on sleep disorders in PBC, being associated with increased sleep latency and earlier wake times [219]. Accordingly, an improvement in pruritus following treatment with either the ileal bile acid transporter inhibitor linerixibat [220] or the peroxisome proliferator-activated receptor (PPAR)-delta agonist seladelpar [221] was accompanied by improvements in sleep quality in PBC patients.

Assuming that sleep disturbances and daytime sleepiness reflect circadian rhythm sleep–wake disorders, an interesting pilot study in PBC patients evaluated the effect of morning bright-light therapy, showing promising improvements in both subjective sleep quality and daytime sleepiness [222].

### 5.3. Can Hepatitis C Virus (HCV) Modulate Sleep?

Up to two-thirds of patients with HCV infection report sleep disturbances [11,223], particularly insomnia, poor sleep quality, low sleep efficiency, increased nighttime awakenings, and excessive daytime sleepiness [224]. These complaints are often associated with psychiatric comorbidities (for example, anxiety, depression, and substance abuse) and medical comorbidities (for example, anemia and hypothyroidism) [11]. However, HCV itself may be neurovirulent, gaining access to the central nervous system through a “Trojan horse” mechanism, whereby infected monocytes cross the blood–brain barrier and replace resident microglia turnover, or via peripherally derived cytokines [225]. These neurotoxic effects of HCV are associated with cognitive impairment and may also contribute to sleep disturbances.

Interferon-based therapy worsens sleep disturbances, which is not the case for direct antiviral drugs [226]. Conversely, viral eradication has been shown to improve sleep quality [227].

A recent study using humanized liver chimeric mice showed that chronic HCV infection alters the expression and rhythmicity of more than 1000 genes in hepatocytes, particularly those involved in key pathways of metabolism, fibrosis, and cancer. How HCV-induced disruptions in hepatocyte circadian rhythms might modulate sleep remains unknown [228].

### 5.4. Sleep Disturbances, Could It Be NeuroWilson?

Wilson’s disease (WD) is an autosomal recessive hereditary disorder of copper metabolism, in which mutations in the ATP7B gene result in impaired biliary copper excretion and subsequent toxic copper accumulation, primarily affecting the liver and the central nervous system [229].

A systematic review reported a 54% prevalence of sleep disturbances among patients with WD, corresponding to a more than 7-fold increased risk compared with controls [230].

WD patients exhibit poor sleep quality, insomnia, excessive daytime sleepiness, prolonged sleep- and REM-onset latencies, shorter total sleep duration, reduced sleep efficiency, and increased wakefulness, with a shift toward greater stage N1 and reduced N2 predominance. The severity of sleep disturbances parallels the severity of neurological symptoms [231]. Interestingly, WD patients show a particularly high prevalence of REM sleep behavior disorder (RBD)—approximately 11% overall, and affecting half of those with neurologic involvement). RBD is a parasomnia in which patients retain motor activity during REM sleep, allowing them to physically enact their dreams [232]. RBD results from lesions or dysfunction in dopaminergic descending pathways and may be the first manifestation of WD. Case reports have shown that RBD symptoms may improve or even resolve following copper chelation therapy [230].

Sleep disturbances in WD may arise from neurologic symptoms that interfere with sleep, such as rigidity, dystonia, tremor, nocturia-related awakenings, or psychiatric comorbidities (e.g., depression). It can also result from direct neurological damage or dysfunction affecting sleep–wake regulation [232,233,234].

## 6. Liver Cirrhosis: How a Deranged Architecture in the Liver Can Impact Sleep Architecture

Sleep disturbances have been reported in 40–85% of patients with cirrhosis, which contrasts with only 10% in the healthy population [235,236,237,238,239,240].

Sleep disorders in liver cirrhosis appear independent of the condition of a chronic disease itself. A small study comparing cirrhotic patients with those with chronic kidney disease found sleep disorders to be 20% more common in cirrhotics. Furthermore, the pattern of sleep disturbances was specific to liver cirrhosis, with poor-quality sleep being associated with delayed bedtime and wake-up times, suggesting a disturbed circadian rhythm [235]. The delayed sleep and wake time, coupled with increased activity during the sleep period, has been corroborated in other studies, and is associated with altered light exposure profiles [241,242,243]. Cirrhotic patients typically have lower light exposure with a higher proportion of exposure to dim light during the day, and increased light exposure across all light intensities at night, amplifying circadian misalignment [243].

In animal models, hyperammonaemic mice present altered expression of circadian rhythm-related genes, such as *Per*, reducing their cyclic expression [244]. Patients with liver cirrhosis seem particularly prone to circadian rhythm deregulation, as cirrhotics display altered melatonin circadian kinetics, with higher diurnal levels and a delay in its rising and nocturnal peak [242,245,246,247]. This can be explained by multiple factors: (a) insufficient light exposure, (b) direct toxicity on the pineal gland by ammonia or other toxins, impairing melatonin production [248], (c) hepatic retinopathy characterized by retinal Muller cells edema impairing retinohypothalamic activity, blunting the suppression of melatonin production by light [249,250], and (d) reduced hepatic metabolism of melatonin into 6-sulfatoxymelatonin [241,251].

Sleep disturbances are common even in compensated patients, and do not seem to correlate with liver enzymes or function, or with the etiology of cirrhosis [237,239]. However, insomnia and poor sleep quality tend to be more pronounced in patients with severe liver insufficiency (Child Pugh Turcotte class C), possibly due to factors such as ascites, diuretics-related nocturia, and pruritus [238,239]. Insomnia is also associated with late-chronotype and psychiatric comorbidities, including anxiety and depression. Compared to controls, cirrhotic patients show prolonged sleep latency, fragmented sleep, less restorative sleep with proportionally shorted REM duration, decreased low-frequency power (suggesting brain dysfunction in slow oscillatory mechanisms during sleep), fewer complete sleep cycles, lower sleep efficiency, and excessive daytime sleepiness, which is often compensated by more frequent and longer daytime naps [235,236,243,247,252,253].

In addition to circadian rhythm disruption, cirrhosis induces sleep disturbances via several other mechanisms. Cirrhotics exhibit impaired homeostatic sleep response, including blunted adenosine receptor activity and density in the brain, which worsens with the presence and severity of hepatic encephalopathy [254]. Cirrhotics also present lower ghrelin levels, a hormone triggered by sleep deprivation [255] and known to promote slow-wave sleep in the second half of the night [256], hence promoting a more restorative sleep. This ghrelin response to sleep deprivation is blunted in patients with cirrhosis [257].

**Figure 1:**
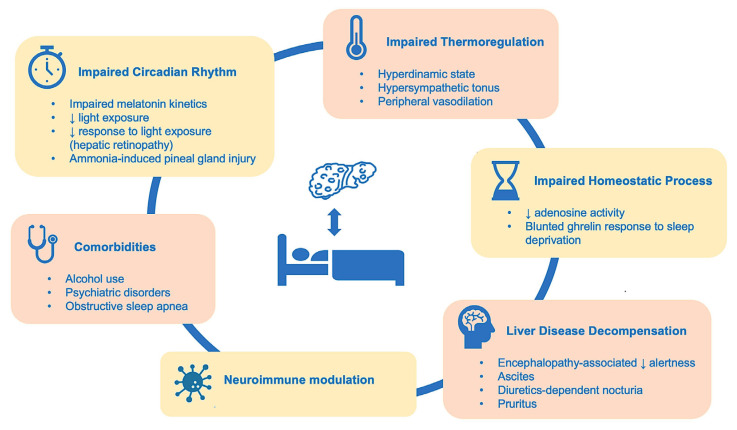
Pathogenesis of liver cirrhosis-induced sleep disturbances.

Impaired thermoregulation in cirrhosis may also impair sleep onset. During sleep onset, melatonin-induced peripheral vasodilation facilitates heat loss, with a subsequent decrease in core body temperature and a decrease in the distal–proximal temperature gradient, hence the aphorism that warm feet precede sleep [258]. The faster the decline in core body temperature, the shorter the sleep latency. Cirrhosis is a hyperdynamic state with basal generalized vasodilation that blunts the peripheral response to melatonin, hampering the acute heat dissipation needed for sleep onset [259] (Figure 1).

Differentiating sleep disturbances from hepatic encephalopathy (HE) can be challenging, as HE is characterized by reversed sleep–wake cycles, with evening restlessness and diurnal sleepiness [260]. Early studies did not find a direct link between sleep quality (assessed by PSQI) or insomnia and HE [235,237], whereas others did find an association between HE and a reduced REM sleep (less restorative sleep), and worsening of sleep quality with decreasing liver function [261,262,263]. Additionally, minimal HE (diagnosed by psychometric cognitive tests) has also been associated with poor sleep quality, longer sleep latency, reduced sleep efficiency, and, most importantly, with excessive daytime sleepiness, which in turn correlates with hospitalization risk for HE [238,239,264]. Indeed, excessive daytime sleepiness has a high negative predictive value (higher than 90%) for HE and is a strong predictor of hospitalization [265]. Interestingly, in the latter study, excessive daytime sleepiness was not associated with sleep disturbances, suggesting a different disease process, with HE being a state of decreased alertness. The treatment of HE with either lactulose or rifaximin has been shown to improve sleep architecture, particularly by increasing REM sleep, although the effects on daytime sleepiness remain inconsistent [266,267].

Hyperammonemia induced by amino acids challenge induced minimal HE in 20% of cirrhotics but not in healthy subjects. The effect on sleep was different in healthy and cirrhotic patients. In the former, it increased NREM sleep duration and slow-wave sleep, whereas in the latter it decreased slow-wave sleep, evoking a less restorative sleep [64,237].

Sleep disturbances and sleep quality are associated with a deterioration in health-related quality of life (HRQoL) [237,238,240]. Sleep disturbances can also have prognostic significance. A study following 150 cirrhotic patients for 9 months showed a dose-dependent increase in mortality with worsening HRQoL, with sleep disturbances increasing mortality risk by 4% [268]. Furthermore, large-scale longitudinal epidemiological studies found a U-shaped association between sleep duration and hepatocellular carcinoma (HCC) incidence, as well as liver-related mortality, with a nadir risk for 7 to 8 h of sleep [269,270]. Moreover, unhealthy sleep patterns and daytime naps (particularly if exceeding 1 h), were associated with a 50% increase in HCC incidence and liver-related mortality [269]. Similar associations were found, with a 50% increased risk for HCC observed with unhealthy sleep, 40% with insomnia, and 30% with snoring [270]. Mendelian randomization studies have provided genetic evidence of a causal effect linking short sleep duration, daytime napping, and insomnia with an increased risk for HCC [271]. One possible mechanism through which sleep disturbances might favor hepatocarcinogenesis is by promoting immunosuppression, and hence decreasing immunosurveillance, in the tumor microenvironment, accelerating tumor growth [272].

We still lack robust evidence on how to manage sleep disorders in cirrhotic patients. General recommendations include the following: (a) the exclusion of HE in patients with excessive daytime sleepiness, (b) the treatment of underlying conditions, such as OSA, hypothyroidism and psychiatric disorders (depression, anxiety), (c) a review of medications that might impair sleep quality, adjusting these if possible (for example, taking diuretics only until lunch time), and (d) the promotion of good sleep hygiene, including regular sleep schedules and avoiding bright light exposure at night [17,68,273].

Specific treatments targeting circadian misalignment have shown mixed results in small trials with cirrhotic patients. Bright light therapy showed promising results in a case report [274]. However, unlike in PBC patients, small trials in cirrhotic patients, either ambulatory compensated or hospitalized and decompensated, treatment with bright light protocols did not improve sleep disturbances [222,275].

Regarding pharmacotherapy, the evidence in patients with cirrhosis is very limited, with few studies, most of which included a small number of participants. Antihistamines, such as hydroxyzine, have significant anti-cholinergic effects, undergo extensive hepatic metabolism, and have a long half-life, which can lead to daytime drowsiness effects, even when taken at bedtime [276]. Antihistamines have the potential to worsen cognitive function, since both histamine and acetylcholine play a role in learning and memory. A small randomized controlled trial involving 25 patients with liver cirrhosis, minimal HE, and sleep impairment found that 25 mg hydroxyzine at bedtime for 10 days improved sleep efficiency by 30% compared to placebo. However, 1 in 17 patients developed an acute episode of HE, which remained irreversible, even after discontinuation of hydroxyzine [277]. As such, hydroxyzine should be used very carefully in patients with liver cirrhosis, particularly decompensated patients. Patients with cirrhosis are particularly sensitive to the sedative effects of benzodiazepines [278]. Benzodiazepines increase the risk of HE and hospitalization due to HE [279,280]. Benzodiazepines also increase the risk for falls in hospitalized patients with cirrhosis [281]. Thus, benzodiazepines should generally be avoided in decompensated cirrhotics. When benzodiazepines are necessary, it is important to take into consideration the hepatic metabolization, preferring drugs with a short half-life and metabolization solely through phase II reactions (conjugation) such as oxazepam, over drugs with both phase II and I reactions (cytochrome metabolization), such as diazepam and midazolam [278]. Zolpidem, a non-benzodiazepine GABA receptor modulator, should also be avoided in patients with decompensated cirrhosis. Data on zolpidem use in cirrhosis is scarce. A small randomized clinical trial involving 52 patients with Child–Pugh–Turcotte class A and B cirrhosis showed that 5 mg of zolpidem nightly for 4 weeks improved sleep efficiency compared to placebo [282]. However, zolpidem is associated with an increased risk of HE and falls in cirrhotics, both of which are linked to high morbidity and mortality [283,284]. The use of melatonin in patients with cirrhosis is physiologically unpredictable, owing to altered melatonin kinetics and reduced hepatic clearance in these patients. Despite this, a small study involving 71 patients with cirrhosis suggested benefits from 3 mg melatonin nightly for 2 weeks, showing an improvement in sleep quality and excessive daytime sleepiness [285]. Finally, liver transplantation seems to be associated with a progressive decline in the prevalence of sleep disturbances [286,287], which seems to be dependent on the etiology of liver disease, with improvements reported for alcohol-associated but not HCV-associated cirrhosis [288]. After liver transplantation, sleep duration and quality tend to increase, while sleep or REM latencies tend to decrease, as well as excessive daytime sleepiness [289].

## 7. Conclusions

More attention should be focused on sleep hygiene and disturbances/disorders as a risk factor for liver disease and as a manifestation of liver disease that warrants proper management.

Sleep as a healthy lifestyle factor is utterly disregarded, even though it has been shown to represent 20% of the association between lifestyle and MASLD. Current American and European guidelines do not address sleep behavior as a risk factor for MASLD except for a brief mention to OSA, nor do they draw recommendations for sleep hygiene as a strategy to treat MASLD. This is in contrast with the profound impact of sleep on MASLD development and also on liver fibrosis progression and even liver-related mortality in these patients.

Similarly, the effect of liver cirrhosis on sleep should be systematically assessed in clinical practice, as it directly impacts quality of life and likely influences outcomes. Excessive daytime sleepiness in patients with cirrhosis should always raise concern regarding impending hepatic encephalopathy.

Sleep disturbances should be considered a critical public health issue, and population-wide education and health policies should prioritize the promotion of good sleep hygiene. Key strategies include aligning bedtime with natural circadian rhythms, adopting early feeding schedules, minimizing exposure to bright light in the evening (such as from TVs and phone screens), promoting light exposure early in the morning, ensuring 7–8 h of sleep per night, and avoiding daytime naps.

Future research on the impact and management of sleep disorders in liver patients is welcomed.

## Figures and Tables

**Table 1:** Summary of sleep disturbances in patients with chronic liver disease.

Liver Disease	Prevalence of Sleep Disorders	Summary of Sleep Disturbances
*MASLD*	33%	U-shape association with sleep duration (nadir of MASLD risk 7–8 H). Steatosis, steatohepatitis, and fibrosis are associated with ↑ sleep latency, poor sleep quality, frequent arousals, and ↓ sleep efficiency. WCUS protects against, but naps promote, metabolic dysfunction and MASLD. Late-chronotype is associated with steatosis and fibrosis. Strong association between OSA and MASLD development and progression.
*Alcohol*	35–90%	Alcohol exposure is associated with: ↓ slow-wave and REM sleep in the first half of the night. ↑ wakefulness and N1 NREM sleep in the second half of the night. Blunted homeostatic recovery following sleep deprivation. Sleep disturbances persist even after 2 years of abstinence and predict future alcohol relapse.
*Primary* *Biliary* *Cholangitis*	50%	↑ sleep latency, poor sleep quality, and short sleep duration. Excessive daytime sleepiness and fatigue as cardinal symptoms. Treatment of pruritus also improves sleep quality.
*Hepatitis* *C Virus* *Infection*	66%	Insomnia, poor sleep quality, ↑ nighttime awakenings, ↓ sleep efficiency, and excessive daytime sleepiness. Potential causes: comorbidities, direct HCV effect, and systemic inflammation. HCV eradication improves sleep disturbances.
*Wilson’s* *Disease*	50%	Sleep disturbances may be the presentation of neuroWilson. Insomnia, ↑ sleep latency, poor sleep quality, ↑ nighttime awakenings, ↓ sleep efficiency, and excessive daytime sleepiness. Particularly high prevalence of RBD (11% overall and 50% if neuroWilson).
*Cirrhosis*	40–85%	Sleep disturbances even in compensated liver disease. ↑ sleep latency, ↑ sleep fragmentation, ↓ sleep efficiency, less restorative sleep, shorter REM and ↓ low-frequency power, ↑ and longer daytime naps. Suspicion of hepatic encephalopathy when excessive daytime sleepiness is noted.

↑, increased; ↓, decreased; MASLD, metabolic dysfunction associated steatotic liver disease; RBD, REM sleep behavior disorder; OSA, obstructive sleep apnea; WCUS, weekend catching-up sleep.

## Data Availability

No new data were created or analyzed in this study.

## Abbreviations

The following abbreviations are used in this manuscript: BMI, body mass index; CPAP, continuous positive airways pressure; DBP, albumin D-element binding protein; E4BP4, E-4 promoter binding protein 4; EDS, excessive daytime somnolence; EEG, electroencephalogram; ESS, Epworth Sleepiness Scale; HCC, hepatocellular carcinoma; HE, hepatic encephalopathy; HLF, hepatic leukemia factor; HRQoL; health-related quality of life; ipRGC; intrinsically photoreceptive retinal ganglion cell; IR, insulin resistance; MASLD, metabolic dysfunction associated steatotic liver disease; MS, metabolic syndrome; NREM, non-REM; PSG, polysomnography; PSQI; Pittsburgh Sleep Quality Index; RBD, REM sleep behavior disorder; REM, rapid-eye movement; SOL, sleep onset latency; STSQS, Sleep Timing and Sleep Screening Questionnaires; SWS, slow wave sleep; OSA, obstructive sleep apnea; T2DM, type 2 diabetes mellitus; TIB, total time in bed; TST, total sleep time; WASO, wakefulness after sleep onset; WCUS, weekend catching-up sleep; WD, Wilson’s disease.
